# Risk factors for postoperative myocardial injury-related cardiogenic shock in patients undergoing cardiac surgery

**DOI:** 10.1186/s13019-023-02312-3

**Published:** 2023-07-06

**Authors:** Xiao-Feng Cheng, Kuo Wang, Hai-Tao Zhang, He Zhang, Xin-Yi Jiang, Li-Chong Lu, Cheng Chen, Yong-Qing Cheng, Dong-Jin Wang, Kai Li

**Affiliations:** 1 Department of Cardio-Thoracic Surgery, Nanjing Drum Tower Hospital Clinical College of Nanjing University of Chinese Medicine, Number 321 Zhongshan Road, Nanjing, 210008 China; 2 Department of Cardio-Thoracic Surgery, Nanjing Drum Tower Hospital Clinical College of Xuzhou Medical University, Number 321 Zhongshan Road, Nanjing, 210008 China; 3 Department of Cardio-Thoracic Surgery, Nanjing Drum Tower Hospital, Chinese Academy of Medical Science & Peking Union Medical College, Number 321 Zhongshan Road, Nanjing, 210008 China; 4 Department of Cardio-thoracic Surgery, Nanjing Drum Tower Hospital, Affiliated Hospital of Medical School, Nanjing University, Number 321 Zhongshan Road, Nanjing, 210008 China; 5 Department of Cardio-Thoracic Surgery, Nanjing Drum Tower Hospital Clinical College of Nanjing Medical University, Number 321 Zhongshan Road, Nanjing, 210008 China

**Keywords:** Myocardial injury, Cardiogenic shock, Cardiopulmonary bypass, Cardiac surgery, Outcomes

## Abstract

**Background:**

Myocardial injury-related cardiogenic shock (MICS) is significantly associated with poor outcomes in patients after cardiac surgery. Herein, we aimed to investigate the risk factor for postoperative MICS.

**Methods:**

We performed a case-control study on 792 patients undergoing cardiac surgery from 2016 to 2019, including 172 patients with postoperative MICS and 620 age- and sex-matched controls. MICS was defined as composite criteria: a cardiac index of < 2.2 L/m^2^/min, arterial lactate levels of > 5 mmol/L at the end of the surgery, a vasoactive-inotropic score of > 40 at the end of the surgery, and a cardiac troponin T (cTnT) level of > 0.8 µg/L on postoperative day 1 (POD1) with an increase of > 10% on POD 2.

**Results:**

A total of 4671 patients who underwent cardiac surgery in our hospital between 2016 and 2019 were included; of these, 172 (3.68%) had MICS and the remaining 4499 did not. For investigating the risk factors, we selected 620 age- and sex-matched controls. In the univariate analysis, MICS was significantly associated with death (*P* < 0.05), extracorporeal membrane oxygenation (*P* < 0.05), continuous renal replacement therapy (*P* < 0.01), and ventricular arrhythmias (*P* < 0.05). Multivariable logistic regression analysis revealed that diabetes mellitus (OR:8.11, 95% CI: 3.52–18.66, *P* < 0.05) and a cardiopulmonary bypass (CPB) time of > 2 h (OR: 3.16, 95% CI: 1.94–5.15, *P* < 0.05) were associated with postoperative MICS. Moreover, long-time administration of preoperative calcium channel blocker (CCB) was associated with a less incidence of MICS (OR: 0.11, 95% CI: 0.05–0.27, *P* < 0.05).

**Conclusions:**

Postoperative MICS is significantly associated with poor outcomes. Diabetes mellitus and long CPB time are associated with MICS. Preoperative CCB administration is associated with less incidence of MICS.

## Background

The incidence of cardiogenic shock after cardiac surgery is approximately 3-9% [1, 2]. Moreover, the incidence of mortality due to cardiogenic shock remains high with more than 15% of patients dying in hospital despite initial successful resuscitation [2, 3]. Therefore, early recognition of postoperative cardiogenic shock is crucial.

Cardiopulmonary bypass (CPB) is a necessary life support during open-heart surgery. An inflammatory response caused by many factors, including CPB, has been known to increase postoperative morbidity and mortality [4, 5]. Inflammation-induced myocardial injury is frequently observed in patients after cardiac surgery and significantly affects their prognosis despite the application of cardioplegic solution intraoperatively [6, 7]. Recent research has demonstrated that inflammation-induced myocardial injury is associated with cardiogenic shock and significantly increases in-hospital mortality and poor outcomes [8]. However, the risk factors of myocardial injury-related cardiogenic shock (MICS) have not been reported. Therefore, we aimed to investigate the risk factors for MICS, which will help clinicians to detect MICS early and improve the prognosis of patients after cardiac surgery.

## Methods

### Study design and settings

This study is a retrospective, observational, convenience sample study of patients who underwent cardiac surgery at a tertiary care center (Nanjing Drum Tower Hospital). This study was approved by the Ethical Committee of Nanjing Drum Tower Hospital. All patients included in this study provided written informed consent [8]. The inclusion criteria were as follows: patients who received aortic valve replacement (AVR), mitral valve replacement (MVR), MVR + AVR, and aortic surgery + AVR, and were > 18 years old. The exclusion criteria were as follows: patients in whom intraoperative Swan − Ganz catheters were not inserted and patients with chronic obstructive pulmonary disease, coronary artery disease, left ventricular ejection fraction of < 35%, preoperative cardiogenic shock, New York Heart Association (NYHA) ≥ III, history of preoperative administration of antiplatelet agents, presence of congenital heart disease, systemic glucocorticoid medication or perioperative glucocorticoid substitution, immunosuppressive medication, pregnancy, extracorporeal membrane oxygenation (ECMO), or preoperative intra-aortic balloon pump (IABP) initiation.

“MICS” was defined as composite criteria. The criteria were consistent with the following conditions: a cardiac index of < 2.2 L/m^2^/min at the end of surgery (T0) [9, 10]; arterial lactate level of > 5 mmol/L at T0 [9]; a vasoactive-inotropic score of ≥ 40 at T0 [9, 11], after correction of all electrolytes and blood gas abnormalities, while adjusting the preload volume to optimal values; and a cardiac troponin T (cTnT) level of ≥ 0.8 µg/L on postoperative day (POD) 1 and an increase of > 10% from POD1 to POD2 [6, 12–14]. The diagnosis of MICS was confirmed when all four criteria were met. A total of 4,671 patients underwent cardiac surgery in our hospital between January 1, 2016, to August 1, 2019. According to the MICS criteria, there were 172 patients with MICS and 4,499 patients without MICS in the included patients. To investigate the risk factors for MICS, 172 patients with MICS and 620 age- and sex-matched controls were selected and analysed. Meanwhile, we identified two to four controls for each case who were matched for sex and age (± 2 years).

### Patient management, sample collection, and biomarker assays

In the operating room, a Swan − Ganz catheter was preoperatively inserted into all study patients. The catheter was inserted through the internal jugular vein; via the superior vena cava; and into the right atrium, right ventricle, and pulmonary artery. A temperature sensor was placed at the front end of the catheter, and then, cold normal saline or glucose solution was injected into the right atrium through the catheter. The cold solution would undergo temperature change after mixing with blood, which was perceived by the temperature sensor. Thus, based on the time of injection and temperature change, the monitor calculated cardiac output and cardiac index [15, 16]. This was performed three times in a row and the average was calculated. Heparin (200–400 U/kg) was administered for anticoagulation in cases of active clotting time of r480 seconds. The CPB circuit was primed with 10–30 g of albumin and 2.5 g of magnesium sulphate injection (concentration: 10%) and 1500–2000 ml of sodium lactate Ringer’s injection. The initial volume of the antegrade cold blood cardioplegia solution (4:1 ratio) was double the volume needed for cessation of all cardiac electrical activity but was never < 1,000 mL. Cardiac arrest was maintained by retrograde infusion of 300 mL of blood cardioplegia solution (8:1 ratio) every 15 min. The CPB flow was adjusted to a target mean arterial pressure (MAP) of 50–80 mmHg, mixed venous oxygen saturation of > 70%, carbon dioxide pressure of 30–40 mmHg, and 32–34 ℃ temperature. At the end of CPB, protamine was used to reverse heparin to a 1:1 ratio, which in turn returned the activated clotting time to preoperative values. Additional doses of protamine may have been required if the activated clotting time was inappropriate. After aortic cross-clamping, vasoactive and inotropic agents, including dopamine (3–10 mg/kg/min), dobutamine (3–10 mg/kg/min), milrinone (0.3–0.6 mg/kg/min), and norepinephrine (0.02–0.10 mg/kg/min), either alone or combined, were given to maintain a MAP of at least 65 mmHg. Extra epinephrine (0.02–0.10 mg/kg min) or vasopressin (0.02–0.07 U/min) was added to vasoactive prescriptions if any cases in the vasopressin group had not reached a MAP of at least 65 mmHg. Extracorporeal membrane oxygenation (ECMO) was initiatively implemented in the operating room if patients had refractory hypotension and met the following conditions [10]: long CPB time (CPB time of > 4 h); need for high-dose vasoactive drugs (vasoactive-inotropic score ≥ 40); cardiac index of < 2.2 L/m^2^/min and MAP of < 60 mmHg; arterial lactate level of > 5 mmol/L; and failure in weaning from CPB.

The vasoactive-inotropic score (VIS) [17] was calculated as follows: dopamine dose (µg/kg/min) + dobutamine dose (µg/kg/min) + [10 x milrinone dose (µg/kg/min)] + [100 x epinephrine dose (µg/kg/min)] + [10,000 x vasopressin dose (U/kg/min)] + [100 x norepinephrine dose (µg/kg/min)].

### Data collection

To investigate the risk factors for postoperative MICS, we examined numerous risk factors, including diabetes, hypertension, atrial fibrillation, chronic renal failure, blood transfusion, calcium channel blocker use, Jinshuibao capsule use, ACEI/ARB use, statin use, type of cardiac surgery, intraoperative AF ablation, CPB time of > 2 h, etc. In addition, we described diabetes in detail, including the type of diabetes (insulin vs. non-insulin-dependent), hemoglobin A1c levels (< 6%, 6-7%, and > 7%) [18, 19], and preoperative fasting blood glucose (< 6.1 mmol/L, 6.1-8.0 mmol/L, and > 8.0 mmol/L) [20]. If CPB time is prolonged, it may result in massive blood transfusions. We collected incidences of massive blood transfusions during cardiac surgery. In Nanjing Drum Tower Hospital, blood products mainly include irradiation apheresis platelet, suspension of red blood cells, virus-inactivated plasma, and cryoprecipitate. Massive blood transfusion is defined as transfusion of ≥ 2 U of irradiation apheresis platelet, ≥ 4 U of red blood cell suspension, ≥ 600 ml of virus-inactivated plasma, and ≥ 8 U of cryoprecipitate in a single operation [21–24].

### Statistical analysis

Statistical analysis was performed using IBM SPSS statistical software (Statistics for Windows, version 25, IBM Corporation, Armonk, NY, USA). Continuous variables are presented as mean ± SD or median with interquartile ranges (IQR). Discrete variables are depicted as frequencies (n, %). Normally distributed continuous variables were evaluated using Student’s t-test. The Mann − Whitney U nonparametric method was used for non-normally distributed continuous variables and the Shapiro − Wilk test for normally distributed continuous variables. Categorical data were compared using the chi-square test or Fisher’s exact test. Covariates reaching statistical significance (*P* ≤ 0.10) in the univariate analysis and those considered clinically relevant were entered into a forward selection multivariable logistic regression model. Then, for each multivariable logistic model, collinearity and calibration were assessed by the value of variance inflation factor (VIF) and Hosmer − Lemeshow test. A *P* value of < 0.05 was considered statistically significant.

## Results

From January 1, 2016 to August 1, 2019, a total of 4,671 patients underwent valvular replacement and valvular + aortic surgery in our hospital. A total of 792 patients met the inclusion criteria, 172 of whom exhibited complicated postoperative MICS, and the remaining 620 age- and sex-matched patients had no postoperative MICS. Detailed demographic data are shown in Table 1.

**Table 1:** Baseline and Characteristic

Variable		Control (n = 620)	MICS (n = 172)		P value	
Age (year)		61.25 ± 13.16	60.45 ± 8.94		0.45	
Gender (male)		282,45.5%	85,49.4%		0.39	
Weight (kg)		64.72 ± 11.82	66.04 ± 14.23		0.22	
Preoperative LVEF (%)		50 ± 11	50 ± 13		0.62	
Preoperative LVDd (cm)		5.8 ± 0.9	5.8 ± 1.1		0.91	
Preoperative cTnT (ug/L)		0.02 ± 0.02	0.02 ± 0.02		0.39	
EuroSCORE		4.82 ± 3.45	4.39 ± 3.29		0.13	
**Previous Medical History**						
Myocardial infarction		3, 0.5%	0		0.36	
Diabetes Mellitus (n,%)		45,7.2%	20,11.6%		0.05	
Hypertension (n,%)		336,54.2%	79, 45.9%		0.02	
Chronic Renal Failure (n,%)		0	3,1.7%		0.02	
Liver diseases (n,%)		51, 8.2%	13,7.6%		0.75	
Previous cardiac operation (n,%)		34,5.5%	6, 3.5%		0.27	
Immunological diseases (n,%)		6, 0.97%	0		0.19	
Atrial fibrillation (n,%)		334, 53.9%	68, 39.5%		< 0.01	
Peripheral vascular diseases (n,%)		13, 2.1%	0		0.05	
Blood products transfusion (n,%)		0	0		—	
Heavily smoking (n,%)		72,11.6%	0		< 0.01	
Excessive alcohol (n,%)		24, 3.9%	3,1.7%		0.24	
Jinshuibao capsule use (n,%)		46, 7.4%	6, 3.5%		0.07	
Aspirin use (n,%)		39, 6.3%	10, 5.8%		0.80	
β-blocker (n,%)		212, 34.2%	67, 39.0%		0.27	
Calcium channel blockers (n,%)		129, 20.8%	11, 6.4%		< 0.01	
ACEI/ARB (n,%)		126, 20.3%	19, 11.1%		< 0.01	
Statin (n,%)		15, 2.4%	15, 8.7%		< 0.01	
**Type of cardiac surgery (n,%)**					0.02	
AVR		21,3.4%	0			
MVR		340, 54.8%	82, 47.7%			
AVR + MVR		202, 32.6%	69, 40.1%			
Aortic operation + AVR		57, 9.2%	21, 12.2%			
Intraoperative AF ablation (n,%)		306, 49.4%	66, 38.4%		0.01	
CPB time (minutes)		161.13 ± 74.83	169.86 ± 51.57		0.08	
ACC time (minutes)		119.84 ± 65.42	127.51 ± 46.44		0.09	

cTnT: Serum cardiac troponin T

MVR: Mitral valve replacement

MIRCS: myocardial injury-related cardiogenic shock

CPB: Cardiopulmonary Bypass

AVR: Aortic valve replacement

LVEF: Left Ventricular Ejection Fraction

LVDd: Left ventricular end diastolic diameter

Patients with MICS had worse outcomes than patients in the control group (Table 2). MICS was significantly associated with in-hospital death (0 vs. 5.81%, P < 0.01), extracorporeal membrane oxygenation (0 vs. 6.98%, P < 0.01), continuous renal replacement therapy (15.32% vs. 55.23%, P < 0.01), and ventricular arrhythmias (7.58% vs. 25.58%, P < 0.01). Patients with MICS had longer ICU stay time (median: 3 days, IQR: 2-7days vs. median: 19 days, IQR: 18–23 days, P < 0.01) and mechanical ventilation time (median 9 h IQR: 5–16 h vs. median: 70 h, IQR: 65–82 h, P < 0.01) compared with the control group. The MICS group had a higher level of cTnT (P < 0.01) during the first 3 PODs compared to the control group.

**Table 2:** Postoperative outcomes

Variable	Control (n = 620)			MICS (n = 172)	P value
Adverse complications					
Death (n, %)		0		10, 5.8%	< 0.01
ECMO use (n, %)		0		12, 7.0%	< 0.01
CRRT use (n, %)		95, 15.3%		95, 55.2%	< 0.01
Ventricular arrhythmias (n, %)		47, 7.6%		44, 25.6%	< 0.01
VIS > 40 more than 4 h (n, %)		149, 24.0%		172, 100%	< 0.01
**Other outcomes**					
Pneumonia (n, %)		8, 1.3%		45, 26.2%	< 0.01
Sepsis (n, %)		0		5, 2.9%	< 0.01
Re-intubation (n, %)		8, 1.3%		20, 11.6%	< 0.01
Re-operation (n, %)		8, 1.3%		14, 8.1%	< 0.01
MV time (hour)		9 (5, 16)		70 (65, 82)	< 0.01
Length of ICU stay (day)		3(2, 7)		19 (18, 23)	< 0.01
**Peripheral arterial cTnT (ug/L)**					
At the end of surgery (T0)		0.53(0.20, 0.83)		0.93 (0.55, 1.29)	< 0.01
At the 3rd hour after surgery (T3)		0.71(0.39, 0.96)		0.98 (0.98, 1.30)	< 0.01
At the 24th hour after surgery (T24)		0.68(0.56, 0.97)		1.17 (0.91, 1.61)	< 0.01
At the 48th hour after surgery (T48)		0.61 (0.47, 0.73)		1.28 (1.11, 1.63)	< 0.01
At the 72nd hour after surgery (T72)		0.51 (0.42, 0.75)		1.11 (0.92, 1.37)	< 0.01

MV: Mechanical Ventilation

VIS: Vasoactive-inotropic Score

Median (interquartile range)

ECMO: Extracorporeal Membrane Oxygenation

CRRT: Continuous Renal Replacement Therapy

In the multivariable analysis, diabetes mellitus (OR: 8.11, 95% CI: 3.52–18.66, P < 0.01) and CPB time of > 2 h (OR:3.16, 95%CI:1.94–5.15, P < 0.01) were associated with MICS after cardiac surgery. Long-time preoperative administration of calcium channel blocker (CCB) was associated with less incidence of MICS (OR:0.11, 95% CI: 0.05–0.27, P < 0.01). Detailed multivariable data are shown in Table 3.

**Table 3:** Multivariable logistic regression

Table 3:				
Variables	Odds ratio	95% CI	P value	
Previous Medical History				
Diabetes Mellitus	8.11	3.52–18.66	< 0.01	
Hypertension	0.96	0.61–1.62	0.97	
Atrial fibrillation	1.15	0.56–2.36	0.70	
Chronic renal failure	0.69	0.37–1.30	0.25	
Jinshuibao capsule use	0.39	0.15–1.01	0.08	
Calcium channel blockers use	0.11	0.05–0.27	< 0.01	
ACEI/ARB use	0.52	0.26–1.01	0.06	
Statin use	3.77	0.56–9.10	0.30	
**Type of cardiac surgery**				
AVR	Reference			
MVR	1.12	0.49–2.54	0.79	
AVR + MVR	1.21	0.55–2.66	0.63	
Aortic operation + AVR	0.76	0.33–1.74	0.51	
Intraoperative AF ablation	0.85	0.48–1.51	0.58	
CPB time > 2 h	3.16	1.94–5.15	< 0.01	

MVR: Mitral valve replacement

ACEI: Angiotensin converting enzyme inhibitors

ARB: Angiotensin receptor blockers

CPB: Cardiopulmonary bypass

AVR: Aortic valve replacement

Diabetes mellitus was associated with MICS, and non-insulin-dependent diabetes (OR:1.3, 95%CI: 1.1–6.9, P = 0.02) had the most significant association. Hemoglobin A1c of > 6% was associated with MICS, of which hemoglobin A1c levels between 6 and 7% (OR: 5.5, 95% CI: 2.1–14.8, P < 0.01) were the most significant. Preoperative fasting blood glucose of > 6.1 mmol/L was associated with MICS (P < 0.01). Intraoperative platelet transfusion of ≥ 2 U (OR: 2.7, 95% CI: 1.2–5.9, P < 0.01) and intraoperative cryoprecipitate transfusion of ≥ 8 U (OR: 3.2, 95% CI: 1.7–6.8, P = 0.02) were associated with MICS. Detailed data are provided in Table 4.

**Table 4:** Univariable and Multivariable logistic for subsequent analysis

Variables			Univariable analysis			Multivariable analysis				
		(n, %)	OR	95% CI	P value		OR	95% CI	P value	
**Diabetes**										
None	727,91.8%		**Reference**				**Reference**			
Non-insulin dependent	56,7.1%		1.8	1.0-3.3	0.04		1.3	1.1–6.9	0.02	
Insulin dependent	9,1.1%		7.8	1.9–31.6	< 0.01		2.3	0.4–12.7	0.35	
**Hemoglobin A1c**										
< 6%	681,86.0%		**Reference**				**Reference**			
6 ~ 7%	85,10.7%		3.9	2.4–6.2	< 0.01		5.5	2.1–14.8	< 0.01	
> 7%	26,3.3%		7.6	3.3–17.2	< 0.01		0.3	0.1–1.3	0.12	
**Preoperative FBG**										
< 6.1mmol/L	604,76.3%		**Reference**				**Reference**			
6.1 ~ 8.0 mmol/L	147,18.6%		4.4	2.9–6.6	< 0.01		3.6	2.0-6.3	< 0.01	
> 8.0 mmol/L	41,5.1%		13.5	6.7–27.2	< 0.01		23.3	7.3–74.5	< 0.01	
**Intra-operative blood transfusion**										
RBC ≥ 4U	248,31.3%		1.4	0.9-2.0	0.06		1.6	0.8–3.4	0.18	
Platelet ≥ 2U	170,21.5%		2.4	1.6–3.5	< 0.01		2.7	1.2–5.9	0.01	
Plasma ≥ 600ml	187,23.6%		1.4	0.9–2.1	0.08		1.8	0.9–3.6	0.09	
Cryoprecipitate ≥ 8U	97,12.2%		2.8	1.8–4.4	< 0.01		3.2	1.7–6.8	0.02	

FBG: fasting blood glucose

RBC: red blood cell

OR: odds ratio

CI: confidence interval

## Discussion

This retrospective study included 4671 patients who received open heart surgery and included 3.68% MICS patients. In this large cohort, 792 patients (172 MICS vs. 620 control) were included in the subsequent analysis. Among these 792 patients, we found that postoperative MICS was significantly associated with poor outcomes. Diabetes mellitus and a long CPB time were associated with MICS. CCB administration was associated with less incidence of MICS. Meanwhile, diabetes mellitus was associated with MICS, of which, non-insulin-dependent diabetes had the most significant association. Hemoglobin A1c levels of > 6% were associated with MICS, of which, hemoglobin A1c between 6 and 7% had the most significant association. Preoperative fasting blood glucose levels of > 6.1 mmol/L, > 2 U of intraoperative platelet transfusion, and > 8 U of intraoperative cryoprecipitate transfusion were associated with MICS.

Myocardial injury caused by cardiovascular surgery with CPB has been proven to be a major contributor to postoperative morbidity and mortality [4, 5]. Moreover, surgery-related trauma, blood transfusion, and blood loss are all important risk factors for perioperative myocardial injury [25, 26]. Hence, myocardial injury is frequently observed in patients who undergo cardiac surgery and significantly affects prognosis [27]. If the CPB time is prolonged, it may result in massive blood transfusions. Patients who receive large transfusions have an increased incidence of complications with an increase in transfusion volume. Patients with massive blood transfusions, particularly platelet and cryoprecipitate transfusion, are prone to transfusion-related circulatory overload, leading to cardiac insufficiency [28, 29]. Thus, CPB time is significantly associated with myocardial injury [8]. Moreover, myocardial injury has been widely recognized as an independent risk factor for cardiogenic shock [6, 12, 13]. This pathophysiological process, called MICS in this study, significantly increases mortality and morbidity [6, 12–14]. Our study demonstrated that long CPB time, ≥ 2 U of intraoperative platelet, and ≥ 8 U of intraoperative cryoprecipitate transfusions are significantly associated with incidences of MICS, and MICS possibly results in poor outcomes.

Diabetes mellitus and elevated blood sugar levels are associated with heart disease. Diabetes mellitus represents an important comorbidity in patients with myocardial injury [30]. Cardiovascular disease is the leading cause of death and disability in people with type 2 diabetes mellitus. The majority of pre-clinical and clinical data demonstrate that diabetic patients are among the most susceptible population to myocardial injury, and cardioprotective effects of ischemic and pharmacological conditioning are compromised in the presence of diabetes [31–35]. Hemoglobin A1c reflects the average blood glucose level of the past three months and is used to evaluate long-term glycemic control and predict the risk of perioperative hyperglycemia. In diabetic and non-diabetic patients, HbA1c is positively associated with a risk of diabetic death, microvascular complications, and myocardial infarction. Therefore, screening for HbA1c levels is crucial when assessing cardiovascular risk [18, 19]. Fasting blood glucose levels reflect the function of pancreatic islet beta cells, generally indicating the secretion function of basal insulin, which is the most commonly used index for detecting diabetes. Furthermore, with the increase in fasting blood glucose levels, the incidence and risk ratio of total cardiovascular events, heart disease and stroke also gradually increase [20]. The mitochondria function and redox state likely have fundamental roles in the increased susceptibility of diabetic patients to myocardial injury [36, 37]. In agreement with these previous studies, we found that patients with diabetes were susceptible to postoperative MICS, especially those with non-insulin-dependent diabetes, hemoglobin A1c levels of > 6%, and preoperative fasting blood glucose > 6.1 mmol/L. Therefore, a preoperative hemoglobin A1c of < 6% and fasting blood glucose levels of < 6.1mmol/L maybe be associated with a reduced incidence of MICS. Normal blood sugar levels are conducive for surgery and recovery and aid in avoiding hypoglycemia (blood glucose < 3.9 mmol/L). Blood glucose levels are routinely monitored every 3–4 h postoperatively, and the insulin dose is adjusted accordingly; moreover, blood sugar levels are maintained within the normal range, and liver and kidney function, and ketone bodies and electrolyte levels are closely monitored.

Of note, we found that CCB administration was a protective factor for MICS. Chouairi et al. reported that the administration of CCBs can reduce myocardial ischaemic reperfusion injury [38]. Whereas, a large cohort study reported that CCB does not provide myocardial protection in patients with myocardial infarction [39]. Our data indicated that CCBs could decrease the rate of MICS. Further studies are needed to investigate the effects of CCBs on MICS in patients undergoing cardiac surgery. Moreover, the angiotensin-converting enzyme inhibitor/angiotensin receptor blockers (ACEi/ARB) had potential protective effects on MICS. The use of ACEI has been shown to reduce short- and long-term mortality in patients with myocardial infarction and heart failure or left ventricular systolic dysfunction. ACEI/ ARBs have been widely recognized as having cardioprotective effects [40]. Therefore, our results were similar to those of previous studies.

In conclusion, postoperative MICS is significantly associated with poor outcomes. Diabetes mellitus and a long CPB time are independent risk factors for MICS. Meanwhile, the administration of CCBs is a protective factor for MICS.

### Study limitations

Our study had some limitations. First, it was conducted at a single institution as an observational study, which is prone to bias. In our study, patients with LVEF of < 35% were excluded. These patients are prone to develop cardiogenic shock. Patients who received coronary artery bypass grafting (CABG) or CABG + valvular surgery had been excluded from our study. These patients usually need a long CPB time. The long CPB time would increase the incidence of severe myocardial injury. We excluded these patients from our study and this could have led to some statistical errors. Given the retrospective design and the limited sample size, the number of controls included per MICS patient was 1:3.6 patients and not 1:4; this could have affected the overall results. Finally, the data come from a single center. It may influence the current results. Future studies should focus on improving the generalization and practicability of our results.

## Data Availability

The datasets generated and/or analyzed during the current study are not publicly available [some patients did not allow us to publish their medical records] but are available from the corresponding author upon reasonable request.
